# The effect of increase in blood glucose level on hearing loss

**DOI:** 10.1016/j.bjorl.2022.06.003

**Published:** 2022-06-15

**Authors:** Guven Akcay, Betul Danısman, Goksun Basaranlar, Pınar Guzel, Narin Derin, Alper Tunga Derin

**Affiliations:** a Hitit University, Faculty of Medicine, Department of Biophysics, Çorum, Turkey; b Atatürk University, Faculty of Medicine, Department of Biophysics, Erzurum, Turkey; c İzmir Demokrasi University, Vocational School of Health Services, İzmir, Turkey; d Kozan State Hospital, Department of Otolaryngology Head and Neck Surgery, Adana, Turkey; e Akdeniz University, Faculty of Medicine, Department of Biophysics, Antalya, Turkey; f Akdeniz University, Faculty of Medicine, Department of Otolaryngology Head and Neck Surgery, Antalya, Turkey

**Keywords:** Brainstem auditory evoked potentials, Diabetes mellitus, Distortion product otoacoustic emission

## Abstract

**Objective:**

Previous studies have shown that hearing function is also vulnerable to the effects of diabetes mellitus which can be shown by brainstem auditory evoked potential and distortion product otoacoustic emission recordings. This study aimed to investigate the changes of brainstem auditory evoked potential and distortion product otoacoustic emission in hyperglycemia and whether there is a relationship between reactive oxygen substances production and hearing deterioration in the rat model.

**Methods:**

25 streptozotocin induced diabetic rats were divided into three groups: control, high blood glucose, and diabetes mellitus. Brainstem auditory evoked potential and distortion product otoacoustic emission were recorded, and thiobarbituric acid reactive substances levels were measured in the brainstem tissue.

**Results:**

At 8 kHz, the latencies of I, II, III, IV, and V brainstem auditory evoked potential waves in high blood glucose and diabetes mellitus groups were elongated, at 16 kHz, only these wave latencies of the diabetes mellitus group were prolonged compared with the control group. A significant decrease was also found in distortion product otoacoustic emission amplitudes at 4, 6, 8, and 10 kHz in the high blood glucose and diabetes mellitus groups compared to the control group. There was a significant increase in thiobarbituric acid reactive substances values due to the increase in blood glucose levels in the high blood glucose and diabetes mellitus groups compared to the control group.

**Conclusion:**

These results suggested that high blood glucose levels may cause hearing impairment not only in the diabetic state but also in the period of hyperglycemia before the onset of manifest diabetes mellitus and reactive oxygen substances may play an important role in the pathophysiology of diabetes mellitus. We suggest that regulating high glucose levels even before the onset of manifest diabetes mellitus may prevent hazardous effects on hearing function.

**Level of evidence:**

Level 3.

## Highlights

• Diabetes is a disease caused by insulin deficiency.
• Lipid peroxidation plays an important role in the pathogenesis diabetes mellitus.
• Diabetes mellitus leads to sensorineural hearing loss.
• Regulation of blood glucose level may prevent hearing loss in diabetic people.

## Introduction

Diabetes Mellitus (DM) is a chronic metabolic disease characterized by hyperglycemia.^1^, ^2^ Inadequate secretion of insulin hormone released from the β-cells of the pancreatic islets or reduced insulin sensitivity leads to DM.^3^ The prevalence of DM has been increasing rapidly all over the world and it is predicted that DM will be one of the most important causes of morbidity and mortality in near future.^4^ According to the World Health Organization (WHO), there were 385 million people with DM worldwide in 2013 and it is expected to be more than 590 million in 2035.^5^ In 2016, diabetes was the seventh leading cause of death and one of the main reasons for diseases such as blindness, kidney failure, heart attack, and stroke.^5^ In animal and human studies, it has been shown that the unfavorable effects of diabetes were strongly associated with the amount of oxidative stress indicated by the production of Reactive Oxygen Species (ROS) which can lead to DNA mutations, changes in the structure and function of proteins, and peroxidation of cell membrane lipids.^6^, ^7^, ^8^, ^9^, ^10^, ^11^, ^12^ Lipid peroxidation caused by free oxygen radicals is one of the important reasons for cell damage and plays a major role in the pathogenesis of diabetes mellitus.^12^ It is known that antioxidant defense systems decrease and ROS increases in these patients.^8^, ^9^ In a study conducted on diabetic rats induced with Streptozotocin (STZ), Thiobarbituric Acid Reactive Substances (TBARS) levels were increased in the brain tissue.^11^

The cochlea and the auditory pathways are also at risk in DM.^13^ Many histopathological changes can occur during the disease such as the decrease in the number of ciliated cells, atrophy of spiral ganglion, and demyelination in the 8th nerve.^14^ Due to these changes, a close relationship between DM and hearing loss has been postulated for so long.^15^, ^16^, ^17^ Studies have shown that Distortion Product Otoacoustic Emissions (DPOAE) and Brainstem Auditory Evoked Potentials (BAEP) can show early damage and dysfunction of the cochlea and efferent nervous system in DM patients and can be used in the early diagnosis of diabetic hearing impairment.^18^, ^19^ It was reported that latencies of these evoked potentials were prolonged in diabetic patients^20^ therefore DM is responsible for dysfunctions of central and peripheral auditory pathways.^21^ Diabetes-related hearing loss is characterized as slowly progressive, bilateral and sensorineural hearing loss.^22^ BAEP is the most common and effective electrophysiologic test used in the clinic to assess the peripheral auditory nervous system and the integrity of the brain subdivisions.^23^ Otoacoustic Emission (OAE) is another important test used in the audiometric assessment of hearing capacity.^24^ DPOAE, which is one of the types of evoked OAE recordings obtained by giving acoustic stimulus, results from the simultaneous delivery of pure sound at two or more frequencies.^25^, ^26^, ^27^ DPOAE is the sound produced by outer hair cells and used to assess the function of the cochlea.^24^, ^26^, ^27^ In this study we aimed to investigate the changes of BAEP and DPOAE depending on the blood glucose levels and whether oxidative stress plays a role in hearing function in the rat model.

## Methods

### Experimental protocol

Three months old female Wistar Albino rats weighing 135–210 g were obtained from the Institutional Animal Care and Use Committee at Akdeniz University. This experimental study was approved by the Laboratory Animals Local Ethics Committee of Akdeniz University (01.09.2014/2014.09.05). Rats were maintained on a standard rat diet and water ad libitum with a 12:12 h dark: light cycle at 23–28 °C. Rats were initially divided into two groups: the first group (Control Group: C Group, n = 10) applied with saline intraperitoneally, the second group (n = 15) was administered a single dose (60 mg/kg) of streptozotocin (STZ, Sigma-Aldrich) intraperitoneally to create DM. Three months after STZ injection, fasting blood glucose values were measured from the blood sample taken from the tip of the tail with a glucometer (Contour Plus, Bayer Healthcare, Germany). According to these values, the second group was divided into two groups as high blood glucose group (HBG, n = 7) and diabetes mellitus (DM, n = 8). Rats with blood glucose levels between 100 and 300 mg/dL were accepted as HBG, and those with over 300 mg/dL were accepted as DM.^28^ At the end of the 4th month, fasting blood glucose levels were measured again and BAEP and DPOAE recordings were taken.

### BAEP and DPOAEs recordings

The BAEP responses were recorded with the Labat Master Version 1.0.0.478. Before audiometric tests, the rats were anesthetized with 50 mg/kg ketamine and 6 mg/kg xylazine intraperitoneally and an otoscopic examination was performed to confirm that all animals had normal external ear canal and tympanic membranes. During electrophysiological recordings, the body temperature of the rats was kept constant at 37 °C. Briefly, an active recording silver needle electrode was affixed to the mastoid of the measured ear, a reference silver needle electrode was attached to the vertex (Cz) and the silver disc electrode used as the ground was placed on the rat's tail. Electrode impedances were less than 5 kΩ. A special probe tip (Medelec Insert earphone Dual 54455/A) was used for acoustic stimulus placed in the external ear canal. BAEP was recorded using 8 kHz and 16 kHz frequency, 60 dB tone-burst stimulus. The potentials were filtered in the 300–3000 Hz band interval and recorded with an average of 200 responses. The severity of the stimulus was measured by decreasing 10 dB after 60 dB to 0 dB and the determination of Vth wave. Minimum sound pressure was accepted as the threshold value. I, II, III, IV, and V wave latencies and I–III, III–V, I–V Interpeak Latencies (IPL) were measured, and comparisons were made between the groups. After BAEP recordings, DPOAE measurements of the rats in the quiet room were taken using the EchoLab OAE device, Labat software 3-times at 1-min intervals. The DPOAE cubic distortion responses (2f1–−f2) were set at 65 dB for f1 and 55 dB for f2 and the f2/f1 frequency ratio was defined as 1.22. The f2 values at 3000, 4000, 6000, 8000, and 10,000 Hz were used as DPOAE parameters and the Signal to Noise Ratio (SNR) of these frequencies were analyzed.^29^

### Brainstem Thiobarbituric Acid Reactive Substances (TBARS) Level Determination

TBARS levels were measured fluorometrically by the method described by Wasowicz et al.,^30^ using 1,1,3,3-tetraethoxypropane as standard. Brainstem tissues were homogenized (Bio-Gen Pro-200) in ice-cold 50 mmoL/L potassium phosphate buffer at pH7. Homogenates were centrifuged at 10.000*g* for 15 min at 4 °C and supernatants were used for the analysis. Then, 50 µL of supernatants were transferred to a tube containing 29 mmoL/L of Thiobarbituric Acid (TBA) in acetic acid (8.75 moL/L) and heated for 1 h at 95 °C in the water bath. The samples were cooled and 25 µL of 5 M HCl was added and extracted with 3.5 mL of *N*-butanol for 5 min. TBARS levels were fluorometrically determined with excitation wavelengths of 532 and emission wavelengths of 547 nm. The results are presented as nmoL/g protein.

### Statistical analysis

Statistical analysis was performed using SPSS 20.0 software. Results are expressed as the means and Standard Error of the Mean (SEM). Statistical significance was set at *p* < 0.05. BAEP, DPOAE, and TBARS wave latencies were analyzed by ANOVA statistical method and the Tukey test was used as a post hoc test.

## Results

**Figure 1:**
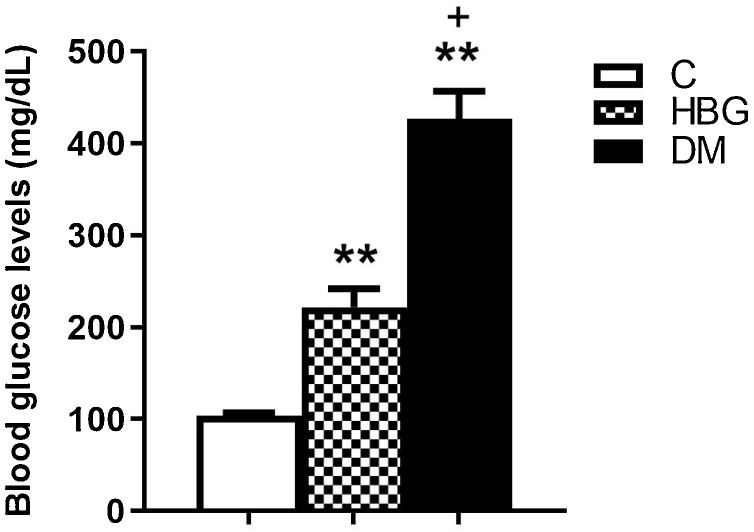
Blood glucose levels in non-diabetic and diabetic rats. Bars represent the group means ± SEM. (C. Control; HBG. High Blood Glucose; DM. Diabetes Mellitus) **p < 0.01 vs. control group. +*p* < 0.05 vs. HBG group.

Blood glucose levels of both HBG and DM groups were found to be significantly higher than the C group (104.33 ± 3.45 mg/dL); blood glucose level of the DM group (421.50 ± 35.93 mg/dL) was also higher than the HBG group (213.83 ± 29.18 mg/dL) (Fig. 1).

**Figure 2:**
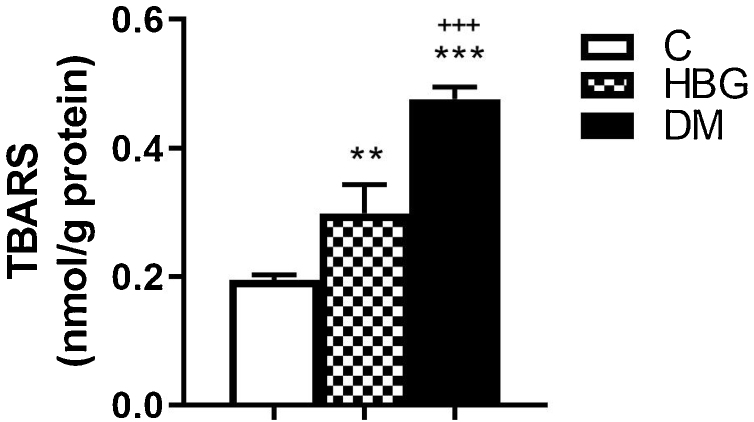
Lipid peroxidation content (TBARS) in the brainstem tissues of all groups. TBARS values are indicated as nmoL/g protein. **p < 0.01 and ***p < 0.001 vs. control group. +++*p* < 0.001 vs. HBG group. Values were expressed as mean ± SEM.

TBARS levels were increased in both HBG (0.30 ± 0.05 nmoL/g protein), and DM (0.48 ± 0.02 nmoL/g protein) groups compared with the C group (0.19 ± 0.01 nmoL/g protein) (Fig. 2). TBARS level of the DM group was higher than the HBG group as well (Fig. 2).

**Figure 3:**
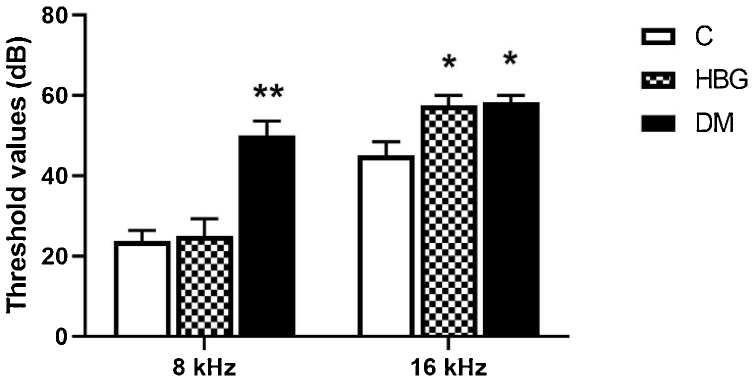
The mean ± SEM of threshold values at 8 kHz. and 16 kHz. *p < 0.05 and **p < 0.01 vs. control group.

In BAEP recordings, at 8 kHz, the threshold value was increased only in the DM group compared to the C group, however the threshold values at 16 kHz were higher in the HBG and DM groups than the C group (Fig. 3).

**Figure 4:**
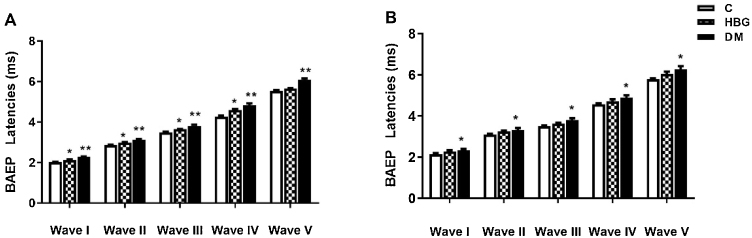
BAEP Waves I, II, III, IV and V of all groups at 8 kHz (A), and 16 kHz (B). Values are expressed as mean ± S.E.M. *p < 0.05. **p < 0.01 vs. control group.

At both 8 kHz and 16 kHz, I, II, III, IV, and V wave latencies of the DM group were significantly higher than the C group (Fig. 4). On the other hand, I, II, III, and IV wave latencies of the HBG group were higher than the C group only at 8 kHz. Although the HBG group demonstrated a prolonged latency of BAEP compared to the control group at 16 kHz, this elongation was not significant.

**Figure 5:**
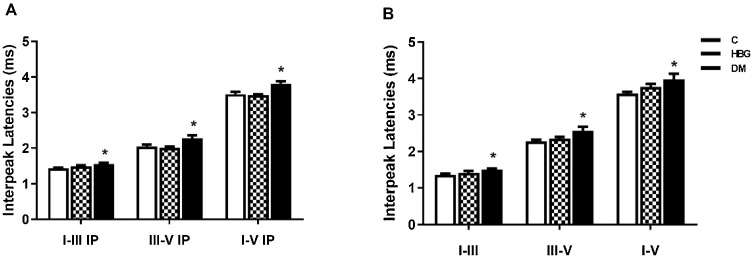
The mean ± SEM of BAEP interpeak latencies at 8 kHz (A), and 16 kHz (B). *p < 0.05 vs. control group.

**Figure 6:**
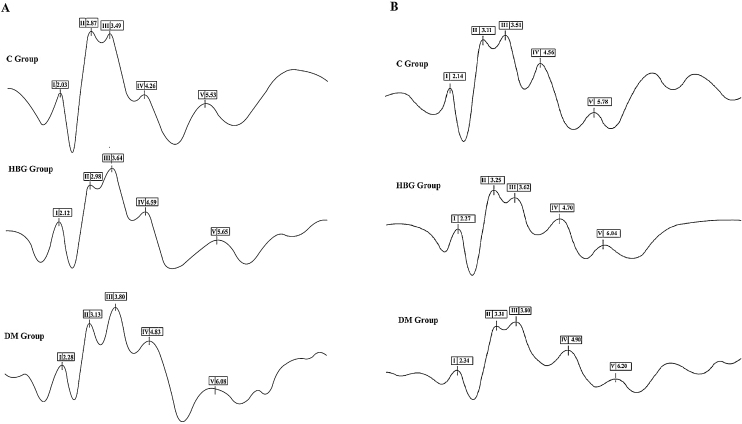
BAEP illustrated traces for all experimental groups at 8 kHz (A). and 16 kHz (B).

There were significant differences between the C and DM groups’ wave I–III, III–V, and I–V interpeak latencies at 8 kHz and 16 kHz, however, no differences were observed between the HBG and the C groups’ interpeak latencies (Fig. 5). Illustrated traces of BAEP recordings in all experimental groups are shown in Fig. 6.

There was a positive correlation between blood glucose level and I, II, III, IV, and V waves latencies and threshold values at 8 kHz. At 16 kHz, a positive correlation was also found between blood glucose level and latency of III wave only. Correlation coefficients between 8 kHz BAEP latencies and blood glucose levels are shown in Table 1, and correlation coefficients between 16 kHz BAEP latencies and blood glucose levels are shown in Table 2.

**Table 1:** Correlation values between blood glucose levels and BAEP latencies at 8 kHz.

Pearson’s R		
Blood Glucose Level, Wave I	y = 0.0005x + 2.1247	0.884^a^
Blood Glucose Level, Wave II	y = 0.0005x + 3.0812	0.763^a^
Blood Glucose Level, Wave III	y = 0.0007x + 3.4563	0.737^a^
Blood Glucose Level, Wave IV	y = 0.0011x + 4.4612	0.766^a^
Blood Glucose Level, Wave V	y = 0.0015x + 5.6617	0.862^a^
Blood Glucose Level, Threshold	y = 0.0357x + 43.602	0.706^a^
Blood Glucose Level, Interpeak I–III	y = 0.0004x + 1.3316	0.576
Blood Glucose Level, Interpeak III–V	y = 0.0006x + 2.2146	0.571
Blood Glucose Level, Interpeak I–V	y = 0.0013x + 3.4613	0.660

a *p* < 0.05 vs. control group.

**Table 2:** Correlation values between blood glucose levels and BAEP latencies at 16 kHz.

Pearson’s R		
Blood Glucose Level, Wave I	y = 0.0007x + 1.9693	0.519
Blood Glucose Level, Wave II	y = 0.0006x + 2.8232	0.535
Blood Glucose Level, Wave III	y = 0.0007x + 3.4514	0.715^a^
Blood Glucose Level, Wave IV	y = 0.0011x + 4.2521	0.503
Blood Glucose Level, Wave V	y = 0.0013x + 5.4002	0.670
Blood Glucose Level, Threshold	y = 0.0717x + 13.57	0.567
Blood Glucose Level, Interpeak I–III	y = 0.0003x + 1.3915	0.473
Blood Glucose Level, Interpeak III–V	y = 0.0006x + 1.9634	0.673
Blood Glucose Level, Interpeak I–V	y = 0.0007x + 3.4315	0.604

a *p* < 0.05 vs. control group.

**Figure 7:**
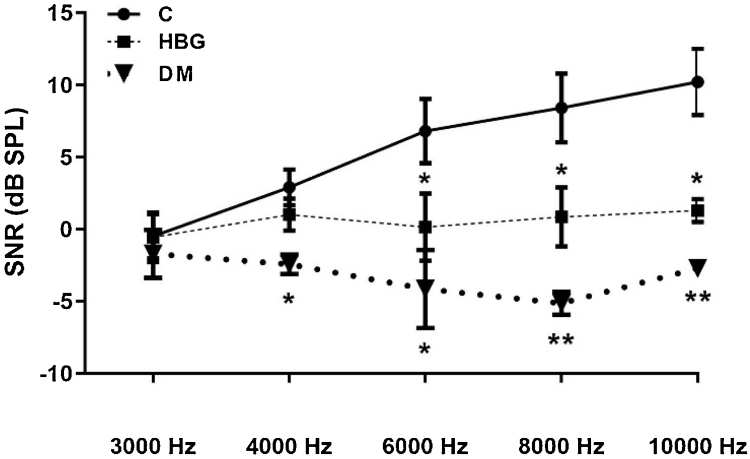
SNR values at 3000, 4000, 6000, 8000, and 10,000 Hz in all groups. *p < 0.05. **p < 0.01 vs. control group. Values were expressed as mean ± SEM.

In Fig. 7, DPOAE values were represented at 3000, 4000, 6000, 8000, and 10,000 Hz. In the DM group, there were significant decreases in the SNR values at 4000, 6000, 8000, and 10,000 Hz when compared to the C group, whereas SNR values at 6000, 8000, and 10,000 Hz in the HBG group were decreased compared to the C group.

## Discussion

Our DM model was conducted according to the studies of Fan et al.^28^ and Buller et al.^31^ In their study Fan et al.,^28^ injected a single dose of 60 mg/kg intraperitoneal STZ and accepted rats as diabetic whose blood glucose levels were higher than 300 mg/dL. With the same study design, Buller et al.^31^ accepted rats as having “high blood glucose levels” between 170 and 300 mg/dL and “diabetic” whose blood glucose levels are above 300 mg/dL. In another study by Domon et al.,^32^ blood glucose concentrations <300 mg/dL were classified as nondiabetic and >300 mg/dL as diabetic, respectively. Based on this information, we used the same experimental protocol and considered rats as having high blood glucose levels between 100–300 mg/dL and DM, those having above 300 mg/dL.

In clinical and experimental studies, BAEP and DPOAE disturbances have been reported in diabetic patients.^33^, ^34^, ^35^ Both diabetic humans and rats experienced slowly progressing hearing loss related to the inner ear and auditory pathway damage.^36^, ^37^, ^38^ Particularly, latencies of waves I, III, and V are prolonged.^31^, ^39^, ^40^, ^41^ In BAEP, I, III, and V waves reflect activity in the acoustic nerve, pons, and midbrain, respectively^29^ which are thought to be important in the early diagnosis of cranial nerve neuropathy associated with DM. Interestingly Abdulkadiroglu et al.^42^ stated in their study that there was no significant relationship between blood glucose levels and the delay of BAEP waves which is contradictory to the results of previously mentioned studies. However, Buller et al.^31^ created an alloxan-induced diabetes model in rats and grouped those with a blood glucose level of 170–300 mg as mild and 300–700 mg as severe diabetes. They showed that the latencies of I, III, V, and the interpeak latencies of III–V and I–V were prolonged.^31^ Di Leo et al.^43^ reported that I, III, and V wave latencies were significantly prolonged in people with diabetes, but the change in interpeak latencies was not significant. Pessin et al.^40^ showed that the I–III interpeak wave latency was prolonged in their study.

In our study, the assessment of the auditory pathway was performed by the BAEP test (at 8 and 16 kHz). We demonstrated that there was a significant prolongation of I, II, III, IV, and V waves’ latencies in both 8 kHz and 16 kHz in diabetic rats, whereas I, II, III, and IV wave latencies were only increased at 8 kHz in HBG group compared to the control. There was no difference in interpeak latencies between the control group and HBG. The threshold value of V. wave was increased only in the DM group compared to the control group at 8 kHz and an increment was also observed in both HBG and DM groups at 16 kHz. Besides, a positive correlation was also demonstrated between blood glucose levels and a delay in BAEP latencies. These findings are substantially concordant with the previous studies^31^, ^40^, ^43^ suggesting damage to auditory pathways not only in manifest DM but also in high glucose levels.

Previous studies have also shown that DM patients had lower DPOAE amplitudes than healthy people.^39^, ^44^ A decrease in DPOAE amplitudes indicates cochlear dysfunction in the early stage of DM. It causes sensorineural hearing loss with a frequency of 3000, 4000, 6000, and 8000 Hz due to damage of the cochlea and efferent nervous system. In our study, we found a significant decrease in the SNR values at 4000, 6000, 8000, and 10,000 Hz in the DM group compared to the control group, whereas a significant decrease was also present in the DPOAE amplitudes in the higher frequency values of 8000 and 10,000 Hz in the HBG group indicating the role of outer hairy cell damage both in manifest diabetic and subclinical hyperglycemic phase of the disease.

It is known that DM causes histopathological changes such as atrophy in the basement membrane of the stria vascularis and basilar membrane in the Corti organ.^45^ Insulin dysregulation and glucose metabolism malfunction are effective in these basement membrane modifications.^46^ Diabetic patients are more vulnerable to diabetes-induced damage to the cochlea and VIII. Nerve.^45^ In some studies of the animal diabetes model, the loss of spiral ganglion cells has been reported.^22^, ^47^ It is thought that this difference may be due to the role of different parts of the Corti organ in frequency transmission.^48^ High-frequency sounds are located near the basilar membrane base of the cochlea, while low-frequency sounds are localized near the basilar membrane apex.^48^ Since the basilar part of the Corti organ contains a frequency of 1–7 kHz, damage to the Corti organ may be caused by diabetes.^45^

There are so many mechanisms postulated for these electrophysiological and histopathological changes in diabetic patients and animals. One of them is that DM may cause a significant increase in lipid peroxidation.^12^, ^13^ Matsunami et al.^12^ have reported significant increases in TBARS levels in the erythrocytes, liver, pancreas, skeletal muscle, and brain of rats with STZ-induced diabetic rats. Our TBARS data, concordant with those previous studies^12^, ^13^ showed increased brain TBARS levels both in the HBG and DM groups suggesting the role of increased oxidative stress in the pathogenesis of DM.

## Conclusion

In this study, BAEP and DPOAE test results revealed that high blood glucose levels themselves, even before the onset of clinically manifest diabetes, in the prediabetic phase, may cause damage to the rat cochlea and auditory pathways. Regulating high blood glucose levels, therefore, may prevent hearing loss which is a well-known complication of diabetes mellitus.

## Conflicts of interest

The authors declare no conflicts of interest.
